# The Identification of Functional Genes Affecting Fat-Related Meat Traits in Meat-Type Pigeons Using Double-Digest Restriction-Associated DNA Sequencing and Molecular Docking Analysis

**DOI:** 10.3390/ani13203256

**Published:** 2023-10-19

**Authors:** Siyu Yuan, Shaoqi Tian, Chuang Meng, Feng Ji, Bin Zhou, Hossam E. Rushdi, Manhong Ye

**Affiliations:** 1 College of Bioscience and Biotechnology, Yangzhou University, Yangzhou 225009, China; 202101223@stu.yzu.edu.cn (S.Y.); mz120211661@stu.yzu.edu.cn (S.T.); mengchuang@yzu.edu.cn (C.M.); 2 Jiangsu Key Laboratory of Zoonosis, Yangzhou University, Yangzhou 225009, China; 3 Institute of Animal Husbandry and Veterinary Medicine, Beijing Academy of Agriculture and Forestry Sciences, Beijing 100089, China; jifengji@sina.com; 4 College of Animal Science and Technology, Yangzhou University, Yangzhou 225009, China; bzhou@yzu.edu.cn; 5 Joint International Research Laboratory of Agricultural & Agri-Product Safety, Yangzhou University, Yangzhou 225009, China

**Keywords:** squabs, fatty acid, intramuscular fat, ddRAD sequencing, candidate genes, molecular docking analysis

## Abstract

**Simple Summary:**

Fat-related traits, such as intramuscular fat content and fatty acid compositions, are important factors affecting meat quality. In this study, we aimed to identify candidate genes affecting fat-related traits in meat-type pigeons. Double-digest restriction-associated DNA sequencing was employed to screen genomic single nucleotide polymorphisms in two meat-type pigeon breeds (the Chinese indigenous Shiqi pigeon and the imported White King pigeon) which demonstrated significant differences in fat-related meat traits. Gene ontology enrichment analysis and pathway analysis were performed on genes harboring genetic variants, and with clear annotations. Nineteen functional genes involved in lipid metabolism were identified as candidate genes that may affect fat-related traits in squabs. A molecular docking analysis further revealed that three non-synonymous amino acid mutations, resulting from the polymorphic sites in three functional genes, could influence the binding properties between the enzymatic proteins and their substrates, which may subsequently alter the catalytic efficiency of enzymes. We suggested that these three genes (*acetyl-CoA acyltransferase 1* and *2*, *acetyl-CoA carboxylase beta*), which encode rate-limiting enzymes in fatty acid synthesis and degradation, were worthy of further investigation to explore their contributions to the discrepancies in fat-related traits in squabs.

**Abstract:**

The Chinese indigenous Shiqi (SQ) pigeon and the imported White King (WK) pigeon are two meat-type pigeon breeds of economical and nutritional importance in China. They displayed significant differences in such meat quality traits as intramuscular fat (IMF) content and fatty acid (FA) compositions in the breast muscles. In this study, we aimed to screen candidate genes that could affect fat-related meat quality traits in meat-type pigeons. We investigated the polymorphic variations at the genomic level using double-digest restriction-associated DNA (ddRAD) sequencing in 12 squabs of SQ and WK pigeons that exhibited significant inter-breed differences in IMF content as well as FA and amino acid compositions in the breast muscles, and screened candidate genes influencing fat-related traits in squabs through gene ontology analysis and pathway analysis. By focusing on 6019 SNPs, which were located in genes with correct annotations and had the potential to induce changes in the encoded proteins, we identified 19 genes (*ACAA1*, *ACAA2*, *ACACB*, *ACADS*, *ACAT1*, *ACOX3*, *ACSBG1*, *ACSBG2*, *ACSL1*, *ACSL4*, *ELOVL6*, *FADS1*, *FADS2*, *HACD4*, *HADH*, *HADHA*, *HADHB*, *MECR*, *OXSM*) as candidate genes that could affect fat-related traits in squabs. They were significantly enriched in the pathways of FA metabolism, degradation, and biosynthesis (*p* < 0.05). Results from molecular docking analysis further revealed that three non-synonymous amino acid alterations, ACAA1(S357N), ACAA2(T234I), and ACACB(H1418N), could alter the non-bonding interactions between the enzymatic proteins and their substrates. Since *ACAA1*, *ACAA2,* and *ACACB* encode rate-limiting enzymes in FA synthesis and degradation, alterations in the enzyme–substrate binding affinity may subsequently affect the catalytic efficiency of enzymes. We suggested that SNPs in these three genes were worthy of further investigation into their roles in explaining the disparities in fat-related traits in squabs.

## 1. Introduction

Meat-type pigeons are an important source of high-quality meat that is characterized by high nutritional and culinary value, excellent edibility, and superb palatability. As the largest country in the world for pigeon farming and consumption, China has an annual production of approximately 680 million squabs (young meat-type pigeons of about 4 weeks old), which accounts for around 80% of global production [1]. The pigeon industry in China has now become the fourth largest poultry industry after that of chicken, duck, and goose. With the growing demand for squab meat, improving meat quality has become one of the major breeding aims in the pigeon industry [2].

In sheep and goats [3], pigs [4], cattle [5], and chickens [6], published results have demonstrated that intramuscular fat (IMF) and fatty acid (FA) compositions are the main factors that affect meat quality (both sensory and technological properties). A desirable IMF content and optimal FA profiles are essential for improving meat qualities regarding meat flavor, tenderness, juiciness, and texture, as well as for meeting the health requirements of the consumers.

In livestock and poultry, the candidate gene strategy is commonly employed to explore the quantitative differences in phenotypic traits. A multitude of functional genes, the majority of which are involved in the biological process of lipid metabolism, have been identified as candidate genes affecting the IMF content of goat meat [7], beef [8], lamb [9], pork [10], chickens [11], and ducks [12]. Compared with studies conducted in these meat-producing animals, relatively less research has been performed in meat-type pigeons [13] and limited research is confined to few articles. *MyoD1* (*myogenic differentiation 1*) and *H-FABP* (*heart-type fatty acid-binding protein*), which play key roles in myogenesis and the intracellular transportation of lipids, respectively, have been studied as candidate genes affecting IMF content in the breast muscles of squabs. Single nucleotide polymorphisms (SNPs) identified in the exonic region or 3′UTR of *MyoD1* [14] and *H-FABP* [15] were demonstrated to be associated with the differences in the IMF content of breast muscles in squabs. Overall, the research conducted in meat-type pigeons is far behind the studies carried out in other livestock and poultry.

Double-digest restriction-site associated DNA (ddRAD) sequencing is a genome complexity reduction technique, which enables the rapid identification of tens of thousands of SNPs at a reasonable cost. In livestock and poultry, RAD sequencing has been successfully employed in genotyping cattle [16,17,18], yaks [19], buffaloes [20], pigs [21], and chickens [22,23,24,25], and has generated corresponding high-density genotyping datasets. However, to our knowledge, there have been no reports on the application of ddRAD sequencing technology for SNP identification in meat-type pigeons. In our previous study, we demonstrated that there were disparities in fat-related traits, such as the IMF content and FA profile, in the breast muscles of two meat-type pigeon breeds that are extensively raised in China, the Chinese indigenous Shiqi (SQ) pigeon and the exotic White King (WK) pigeon [26]. However, research on the genetic variations within and among the two pigeon breeds is still insufficient. Therefore, this study was conducted for the genome-wide identification and annotation of SNPs in SQ and WK meat-type pigeons using ddRAD sequencing, followed by the identification of candidate genes that could potentially affect fat-related traits in squabs.

On the other hand, despite the fact that considerable research has been performed to screen candidate genes associated with fat-related traits in livestock and poultry, few studies have further investigated the relationship between the function of proteins encoded by candidate genes and the SNPs within them. Most research has been unable to elucidate the effects of these genetic variations on protein functionality. Molecular docking is a computational approach that has been extensively used to assess the interactions between two molecules, such as a macromolecular receptor (an enzyme or protein) and a small ligand [27], and predict their binding conformation and affinity [28]. To address the research limitations mentioned above, we also performed in silico molecular docking analysis to study the enzyme–substrate interactions, which will help to predict the potential impacts of altered amino acids (AAs) on the binding properties of the enzymatic proteins to its substrate and thus on their enzymatic activities. The results obtained in this study created a theoretical map between genetic variations and enzymatic properties regarding substrate–enzyme interactions, which enabled us to gain a deeper understanding of the differences in fat-related meat traits that are associated with genetic variations in functional genes.

## 2. Materials and Methods

### 2.1. Sample Collection and Phenotyping

A total of 12 four-week-old squabs from two meat-type pigeon breeds, Shiqi (SQ) pigeons and White King (WK) pigeons (half female and half male per breed), were used in this study. All birds were reared under the same management system and processed in facilities operated by Yangzhou University (permit No. 202202169). About 1 mL of blood was collected from the wing vein and withdrawn into a centrifuge tube coated with 80 μL of 15 mg/mL sodium ethylenediamine tetraacetate (EDTA-Na_2_). The anti-coagulated blood samples were stored at −80 °C for subsequent DNA extraction.

The squabs were slaughtered via cervical dislocation. The breast muscles of both sides were dissected from the carcasses. After the removal of visible adipose and connective tissues, muscle samples were used for the determination of physical and chemical traits related to meat quality, including pH24 (pH value 24 h postmortem), WHC (water-holding capacity), WBSF (Warner–Bratzler shear force), IMF (intramuscular fat) content, and the fatty acid composition of IMF. These phenotypic traits were measured according to the previously described protocols [13]. The contents of free amino acids (AAs) in the breast muscle of squabs were analyzed using an Automatic Amino Acid Analyzer (L-8900, Hitachi Ltd., Tokyo, Japan). Firstly, the minced breast muscle sample was dried. Then, 0.1000 g of the dried sample was accurately weighed and placed in a thick-walled test tube, followed by the addition of 15 mL of 6 M hydrochloric acid (HCl). The tube was filled with nitrogen gas, sealed, and placed in an oven at (110 ± 1) °C, where the sample was hydrolyzed for 24 h. After cooling and thorough mixing, the contents were transferred to a 100 mL volumetric flask and diluted to 100 mL with ultrapure water. After filtering with a 0.22 µm filter membrane, a measure of 200 μL was taken and vacuum freeze-dried. The dried sample was dissolved with 1 mL of 0.02 M HCl and analyzed for the contents of free AAs.

### 2.2. Construction and Sequencing of the ddRAD Sequencing Library

The procedures described by Peterson et al. [29] with minor modifications were used for the preparation of the reduced representation ddRAD sequencing library. Blood samples were used to extract genomic DNA (gDNA) using a gDNA extraction Kit (Tiangen Biotech, Co., Ltd., Beijing, China). The concentration of gDNA was evaluated using a NanoDrop^TM^ 2000 spectrophotometer (Thermo Fisher Scientific Inc., Waltham, MA, USA) and the quality of the gDNA was assessed through electrophoresis on 0.8% agarose gels. The qualified DNA samples were processed for library construction.

In brief, the gDNA samples were first treated with RNase A (final concentration of 0.05 µg/µL) for 1 h at 37 °C to remove potential RNA contamination. Then, around 500 ng of gDNA (adjusted to 100 ng/µL) from each sample was digested to completion by two endonucleases *Eco*RI and *Nla*III (New England Biolabs, Ipswich, MA, USA), which recognizes the 6-nucleotide rare-cutting sequence 5′-G|AATTC-3′ and the 4-nucleotide frequent-cutting sequence 5′-CATG|-3′, respectively. The double-digestion was performed at 37 °C for 4 h in a 50 μL reaction, followed by a heat-inactivation step at 65 °C for 20 min. The products obtained from double digestion were cleaned with a 1.8× ratio of AMPure XP beads (Agencourt Bioscience Corporation, Beverly, MA, USA), followed by ligation with the P1 adapter (containing barcodes unique to each sample) with an *Eco*RI overhang and the P2 adapter with a *Nla*III overhang.

After the ligation step, each sample was heat-inactivated at 65 °C for 20 min, and equal amounts of the individually barcoded samples were pooled together. The obtained pooled DNA samples were separated via 2% agarose gel electrophoresis followed by a size-selection step. DNA fragments between 300 and 400 bp were gel-excised and purified using a Min Elute Gel Extraction Kit (Qiagen, Hilden, Germany). In order to enrich the adapter-ligated DNA fragments, purified products were used as templates and a 12-cycle PCR procedure was run using the P1/P2 amplification primers. Indexed PCR products were purified using the QIA quick PCR Purification Kit (Qiagen, Hilden, Germany). The resulting libraries were quantified using a Qubit 2.0 Fluorometer (Invitrogen, Carlsbad, CA, USA) and a check of the library fragment size was performed on the Agilent Technologies 2100 Bioanalyzer (Agilent Technologies, Santa Clara, CA, USA). Finally, 10 pM of equimolarly pooled qualified libraries were pair-end sequenced on an Illumina HiSeq PE150 platform in Genepioneer Biotechnologies, Co., Ltd., Nanjing, China, yielding pair-end reads of around 300 bp each.

### 2.3. Quality Filtering and Mapping to the Reference Genome

The bioinformatics analyses were performed by technicians of Genepioneer Biotechnologies, Co., Ltd., Briefly, FastQC v0.11.9 software was used to check the initial quality of the reads. Raw reads that lacked the correct barcodes, were polluted by adapter sequences, or had more than 40% of base calls with Phred quality scores lower than 20 were removed from the dataset. Trimmomatic v0.39 was used to trim the adaptor sequences [30]. Reads with lengths of less than 50 bp were removed. Stacks v2.53 software was used to discard reads without the cut sites for restriction enzymes at the end and quality-filtered reads using a sliding window method (15% of read length) implemented in the “process_radtags” utility [31]. After being subjected to demultiplexing, quality checks, and adapter trimming, the quality-filtered reads of each sample were individually aligned to the reference genome of *Columba livia* (GCA_001887795.1) with BWA (v0.7.5) [32]. Default parameters were used, allowing up to 4 mismatches and one gap when aligning reads to the published pigeon genome.

### 2.4. Detection and Annotation of SNPs

This procedure has been described in our previous report [20]. Briefly, the generated sequence alignment map (SAM) files were converted into sorted and indexed binary alignment map (BAM) files using SAMtools (v1.6). Duplicate reads were removed using the Picard tool (v1.103; http://broadinstitute.github.io/picard, accessed on 21 February 2023). The indexed BAM files of all the 12 animals were merged into a single BCF file using bcftools v0.1.18, which was further converted into a variant calling format (VCF) file. SNPs were preliminarily filtered using the Genome Analysis Tool Kit (GATK) VariantFiltration function by following the GATK Best Practices recommendations [33]. Further filtering of the bi-allelic SNPs was carried out using vcftools v01.16 [34], and the variants were filtered based on the following criteria: SNP missing rate across all samples = 0.2; minor allele frequency ≥ 0.01; minimum Phred quality score for each bases ≥ 10. Gene ontology (GO) and KEGG pathway analyses were performed using DAVID Bioinformatics Resources (https://david.ncifcrf.gov/tools.jsp, accessed on 19 June 2023), a web service for functional enrichment analysis and functional annotations of gene lists.

### 2.5. In Silico Molecular Docking

The impacts of non-synonymous AA mutations on the binding properties of proteins to their substrates were studied through molecular docking. The binding affinity of proteins to the ligand was analyzed through the AutoDock Vina program 1.5.7 (http://vina.scripps.edu/, accessed on 6 July 2023) [35]. The secondary structure and 3D conformation of the proteins (ACAA1, ACAA2, ACACB, ACAT1, ACOX3, FADS1, HADHA, HADHB, MECR, and OXSM) were predicted through the homology modeling network synthesis server (https://swissmodel.expasy.org/, accessed on 28 June 2023). AutoDock Tools (v1.5.7) were used to add polar hydrogen to the protein, compute the Gasteiger to calculate the distribution of atomic charges, assign the AD4 type to atoms to optimize the protein structures for docking, and transfer the files from the .pdb format to the .pdbqt format for docking. The 3D structures of protein ligands were downloaded from published articles in the mol2 format, which were then converted to the .pdb format using OpenBabel (v2.4.1). Consurf (http://consurf.tau.ac.il/, accessed on 3 July 2023) was used to predict the active sites of these proteins. The docking of the flexible ligand to the rigid receptor was performed. The docking simulation was run with an exhaustiveness level of 9. After docking, the conformation of ligands with the strongest affinity to the receptor in the output of AutoDock Vina was used for subsequent analysis. The protein–ligand interactions were analyzed using LigPlot+ (v2.2.8), and the generated output files were visualized and plotted using PyMOL (Version 2.5.5).

### 2.6. Statistical Analysis

The results of the meat quality-related traits are presented as mean ± standard error, with 3 replicates for each gender and for each breed. The statistical analysis was conducted with SPSS Statistics V22.0 (IBM Corporation, Armonk, NY, USA). An unpaired two-tailed *t* test was used for comparisons. Differences were considered statistically significant at *p* < 0.05.

## 3. Results

### 3.1. Physical and Chemical Properties of Breast Muscles

In this study, we compared the fat-related traits of the breast muscles of squabs from two meat-type pigeon breeds (Table 1). Compared with the female WK squabs, the female SQ squabs were significantly higher in such traits as IMF content (*p* = 0.004), total ω-6 FA content (*p* = 0.014), and total PUFA (polyunsaturated fatty acid) content (*p* = 0.016), especially C18:2 (*p* = 0.015) and C20:5 (*p* = 0.006). In the meantime, the WK female squabs had a significantly higher total content of MUFAs (monounsaturated fatty acids) than that of the female SQ squabs (*p* = 0.026), especially C16:1 (*p* = 0.006) and C18:1 (*p* = 0.045). The male squabs of the two breeds exhibited similar trends in fat-related traits to the female squabs. For example, the male SQ squabs had significantly higher levels of C18:2 (*p* = 0.009) and total ω-6 FA content (*p* = 0.008) than the male WK squabs, while the male WK squabs displayed significantly higher levels of C16:1 than the male SQ squabs (*p* = 0.046). Additionally, the male SQ squabs had significantly higher C16:0 contents (*p* = 0.034) and P/S ratios (the ratio of polyunsaturated fatty acids to saturated fatty acids) (*p* = 0.001) than the male WK squabs. Our results indicated that the SQ and WK squabs, especially the female squabs, differed in their accumulation of different types of FAs. The SQ squabs showed a significant advantage in the accumulation of PUFAs, while the WK squabs favored the accumulation of MUFAs. Overall, the SQ squabs exhibited a relatively stronger ability to accumulate fat in the breast muscles than the WK squabs.

Our results (Table 2) showed that the breast muscles of the squabs were rich in AAs, including seven essential amino acids (EAAs) and ten non-essential amino acids (NEAAs). When the breast muscles from the female squabs of the two breeds were compared with each other, no significant differences were observed in the tested items, except that the female SQ squabs had a significantly higher EAAs/NEAAs ratio in their breast muscles than the female WK squabs. Compared with the male SQ squabs, the male WK squabs had significantly higher levels of umami AAs (including Asp, Glu, Gly, and Ala) (*p* ≤ 0.048) and two branched-chain AAs (BCAAs), Val and Leu (*p* ≤ 0.014). The significantly higher content of umami AAs in the male WK squabs than that in the SQ squabs indicated more savory and palatable characteristics of the breast muscles. These results confirmed that the breast muscles from the SQ and WK squabs displayed significant differences in their physicochemical characteristics related to meat quality.

### 3.2. ddRAD Sequencing and SNP Discovering

In this study, a total of 11.19 million processed reads with an average read length of 147 bp were generated from 12 samples of squabs after removing reads without full barcodes and those without enzyme cut sites. The average read number was 9.33 million per sample, varying from 5.78 million to 13.45 million. Across all the samples, the sequencing quality score of 20 (Q20) and 30 (Q30), which represent an error rate of 1% and 0.1%, respectively, was 92.71% and 84.34%, respectively, indicating that the sequencing quality was good (details of the sequencing output are shown in Supplemental Table S1). After adapter removal, quality checking, and demultiplexing, the retained sequencing reads were aligned against the reference pigeon genome. The mean mapping rate was 98.6% (ranging from 98.3% to 98.9%), resulting in an average sequencing depth of 1.22× (ranging from 0.76× to 1.77×) across the genome and a mean coverage depth of 6.97× (Supplemental Table S2).

### 3.3. Distribution and Annotation of SNPs

**Figure 1:**
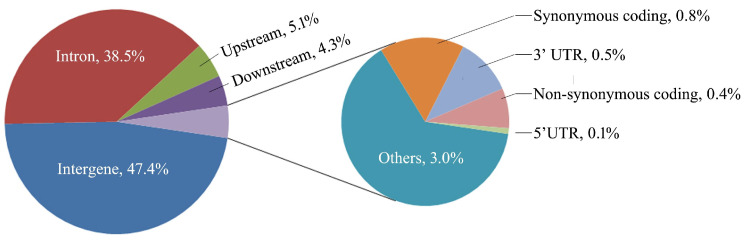
Distribution of SNPs in the genome.

The basic information on the distribution of SNPs that were detected in this experiment on the genome is shown in Figure 1. Among them, there were 4292 SNPs located in the gene 5′-UTR and 3′-UTR; 293,091 SNPs were located in introns; a total of 8725 SNPs were located in the gene coding region.

**Figure 2:**
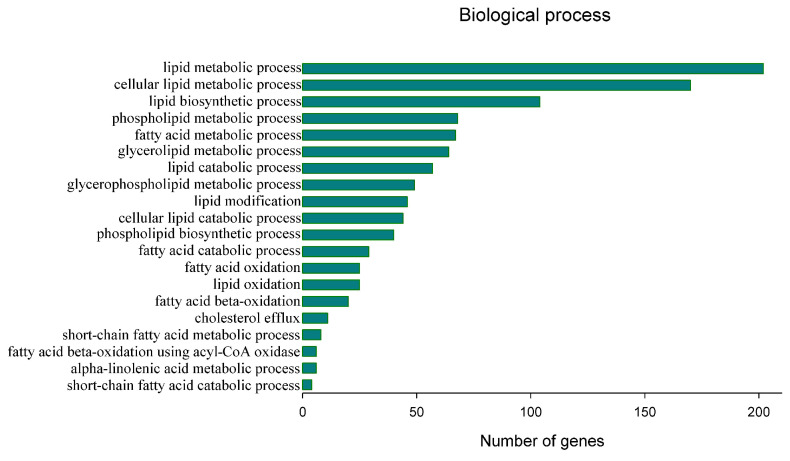
Top 20 GO BP terms related to the lipid-associated metabolism.

Genes harboring SNP sites were annotated, and we focused on selecting 6019 SNPs which were located in genes with clear annotations and had the potential to induce changes in the encoded proteins. These selected SNPs included polymorphic loci in 5′- and 3′-UTRs, the loss and acquisition of start or stop codons, mutations in splice donor or acceptor sites, as well as SNPs causing synonymous and missense mutations in AAs. Collectively, these SNPs came from a total of 2526 functional genes. A GO analysis of these genes showed that a total of 224 genes were involved in biological processes (BPs) related to lipid metabolism, with 217 genes participating in the top 20 BPs related to lipid metabolism, mainly including the biosynthesis of lipids, the oxidation of FAs, and metabolism related to glycolipids, phospholipids, and cholesterol (Figure 2).

A KEGG pathway analysis further revealed that the significantly enriched pathways that these 217 genes were involved in included (1) the fatty acid metabolism pathway (clv01212: fatty acid metabolism, *p* = 9.29 × 10^−4^), in which 18 functional genes were involved (*ACAA2*, ACADS, *FADS1*, *HACD4*, *ACSBG2*, *FADS2*, *ACAT1*, *HADHA*, *HADHB*, *ACOX3*, *ACSBG1*, *OXSM*, *ACSL1*, *ELOVL6*, *HADH*, *ACSL4*, *MECR*, and *ACAA1*); (2) the fatty acid degradation pathway (clv00071: fatty acid degradation, *p* = 0.034), including 12 functional genes (*ACAA2*, *ACSL1*, *ACADS*, *ACSBG2*, *HADH*, *ACSL4*, *ACAT1*, *HADHA*, *ACAA1*, *HADHB*, *ACOX3*, and *ACSBG1*); and (3) the fatty acid biosynthesis pathway (clv00061: fatty acid biosynthesis, *p* = 0050), including six functional genes (*ACSL1*, *ACSBG2*, *ACACB*, *ACSL4*, *OXSM*, and *ACSBG1*).

**Figure 3:**
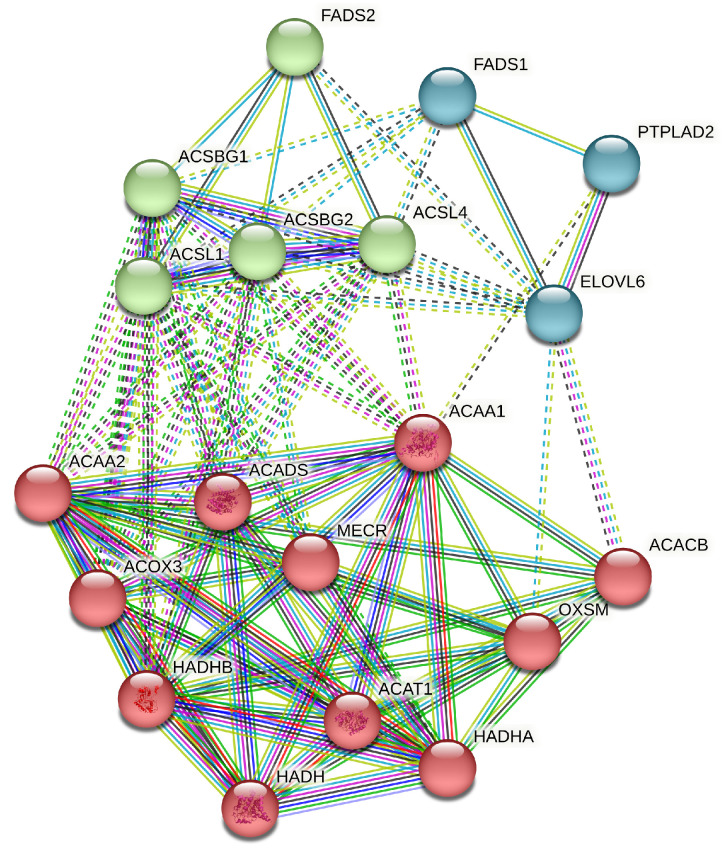
Summary view of protein–protein interactions (PPIs) of 19 genes involved in three significantly over-presented pathways (analyzed via String: background, *Columba livia*; minimum required interaction score, 0.7; number of clusters, 3; average local clustering coefficient, 0.527; PPI enrichment *p*-value < 1.0 × 10^−16^).

In total, 19 functional genes were involved in these three significantly enriched KEGG pathways, and the SNPs detected on them mainly caused missense mutations (Table 3) or were located in the 3′ UTR. These genes can be further studied as candidate genes that affect fat-related meat quality traits in meat-type pigeons. Their protein–protein interactions (PPIs) were analyzed using STRING (Figure 3), and the network clusters of PPIs are shown in Table 4. Most of the clusters were associated with FA metabolism, and two were involved in the degradation of BCAAs (Val, Leu, and Ile).

### 3.4. In Silico Docking Results

**Figure 4:**
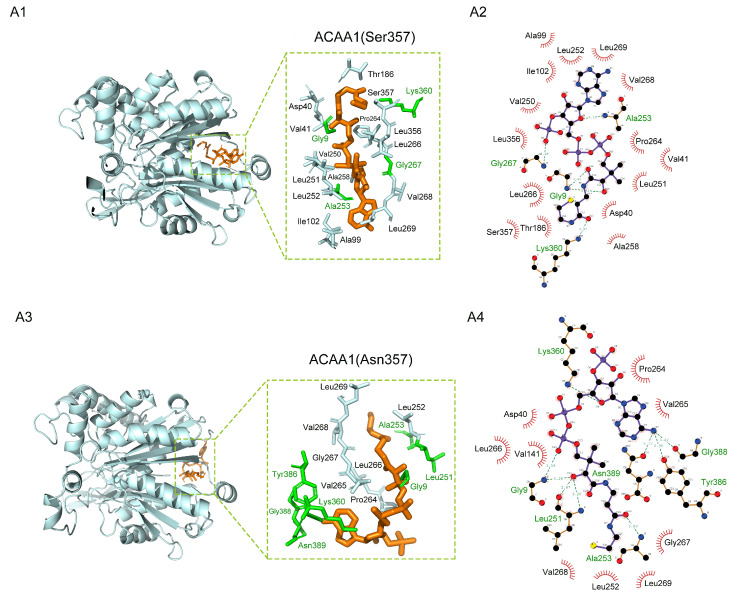

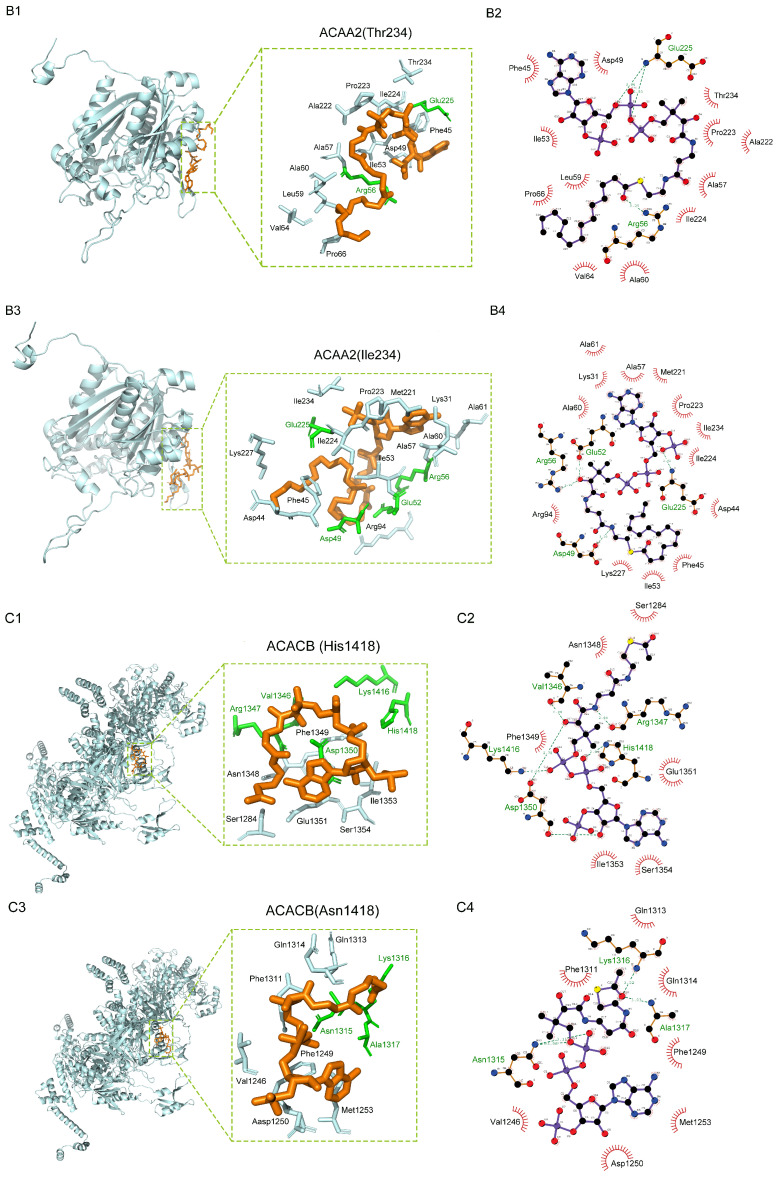
Three-dimensional view of the substrate in complex with the protein after docking. The ligands are represented in orange. In (**A1**,**A3**,**B1**,**B3**,**C1**,**C3**), amino acids labeled in pale cyan form hydrophobic interactions with the ligand, while amino acids labeled in green form hydrogen bonds with the ligand. (**A2**,**A4**,**B2**,**B4**,**C2**,**C4**) are the 2D diagrams showing the hydrophobic interactions and hydrogen bonds formed between amino acids and the ligand. Hydrogen bond is represented by dashed green lines. Hydrophobic interactions are represented by half circles. The resolved center coordinate is shown in Supplemental Table S3.

As shown in Figure 4A, ACAA1(Ser357) and ACAA1(Asn357) had common hydrophobic interaction sites, which included Asp40, Leu251, Leu252, Leu266, Val268, and Leu269. They also shared Gly9, Ala253, and Lys360 as the residues involved in the formation of hydrogen bonds with the ligand. The missense mutation of the 357th AA on the protein resulted in alterations in the interactive residues, as well as the type of interactions between the ligand and the active site of the protein. In ACAA1(Ser357), Gly267 formed a hydrogen bond with the ligand, while in ACAA1(Asn357), Gly267 interacted with the protein through hydrophobic interactions. In ACAA1(Asn357), Tyr386, Gly388, and Asn389 formed extra hydrogen bonds with the ligand. The affinity scores of ACAA1(Ser357) and ACAA1(Asn357) to the ligand were −7.9 kcal/mol and −5.8 kcal/mol, respectively.

Similarly, in ACAA2(Thr234) and ACAA2(Ile234) (Figure 4B), Arg56 and Glu225 formed hydrogen bonds with the ligand. Phe45, Ile53, Ala57, Ala60, Pro223, and Ile224 were involved in the hydrophobic interactions with the ligand. The interactions between Asp49 and the ligand were hydrophobic interactions in ACAA2(Thr234) and a hydrogen bond in ACAA2(Ile234). The binding affinity scores of ACAA2(Thr234) and ACAA2(Ile234) to the ligand were −5.9 kcal/mol and −5.3 kcal/mol, respectively. In ACACB (Figure 4C), the switch of basic His to acidic Asn impacted the conformation around the mutated residue. As a result, there were five hydrogen bonds in ACACB(His1418) and only three in ACACB(Asn1418). The affinity scores of ACACB(His1418) and ACACB(Asn1418) to the ligand were −5.3 kcal/mol and −5.1 kcal/mol, respectively. Our results showed that these non-synonymous AA mutations in ACAA1, ACAA2, and ACACB could lead to changes in the non-covalent interactions between the protein receptor and its ligand. These changes were likely to impact the binding and catalysis of the protein towards substrates, thereby affecting the enzymatic activities.

## 4. Discussion

In the research of meat quality traits, there is a comparative lack of studies on the inter-species differences among different meat-type pigeon breeds and the screening of functional genes with potential impacts on meat quality traits. The majority of the currently available literature focuses on studies of nutritional requirements [36,37], feeding strategies [38,39], and the effects of dietary components on the growth performance of meat-type pigeons [40,41,42]. In the current study, we analyzed the IMF content, FA composition, and AA composition of the breast muscles from 4-week-old squabs of two meat-type pigeon breeds. Our results confirmed that the SQ squabs and WK squabs displayed significant differences in these meat-quality-related traits, which were consistent with previous reports [26,43]. Our main objective focused on screening functional genes that could be potentially related to these disparities in meat quality traits.

Based on the results obtained from the ddRAD approach, we primarily identified 19 functional genes affecting the fat-related traits of the breast muscle in squabs through pathway analysis. Among these genes, the majority have been reported to be associated with fat-related meat quality traits in livestock or poultry, while some have not been clearly documented on their roles in fat deposition so far. Studies regarding the identification of these candidate genes associated with fat-related traits in pigs, cattle, sheep, and buffalo are summarized in Supplemental Table S4.

In chickens, FADS1 and FADS2 are two rate-limiting enzymes involved in the conversion of ALA (α-linolenic acid) to DHA (docosahexaenoic acid). The latter is one of the most important beneficial n-3 PUFAs for consumers. The up-regulated expression of *FADS1* and *FADS2* was associated with a healthier FA profile of the breast muscles in slow-growing chickens [44] and in rutin-supplemented chickens with improved meat quality [45]. Elongase, ELOVL6, catalyzes the rate-limiting reaction in the long-chain FA (12–16 carbons) elongation cycle in the de novo lipogenesis of saturated and monounsaturated FAs with 18 carbons. At the transcriptional level, the expression of *ELOVL6* in chickens was found to be related to the content of abdominal fat, while the expression of *ACSL1* and *FADS2* was associated with IMF content in the breast muscles [46]. The FA deposition-related enzyme, ACAT1, plays important roles in cholesterol homeostasis. It utilizes long-chain fatty acyl-CoA and cholesterol as substrates to form cholesteryl esters that coalesce into cytosolic lipid droplets [47]. The increased expression of *ACAT1* and *ACOX3* was demonstrated to be associated with relatively higher IMF in the pectoralis major of Arbor Acres chickens at hatching [48]. ACSBG2, which catalyzes the conversion of long-chain FAs to their active form acyl-CoAs for both lipid synthesis and beta-oxidation, was identified as a key gene involved in the adipogenic differentiation of chicken preadipocytes [49]. Furthermore, in laying hens of different ages, HADH, ACAA2, HADHA, and ACSL1 were differentially expressed proteins associated with a disparity in FA compositions [50].

Overall, findings regarding the identification of these candidate genes, associated with fat-related traits in livestock (pigs and cattle, especially) and chickens, have been accumulated. However, few studies have been conducted on meat-type pigeons, leaving a significant knowledge gap. Furthermore, to our knowledge, most of these studies mainly focused on differential gene expression or phenotype-related association analysis. Few researchers have explored the physiological and biochemical significance underlying these associations. In this study, we further investigated the potential impacts of altered AAs on the binding properties of the enzymatic protein to its substrate using molecular docking analysis.

In the substrate–enzyme system, conformational dynamics are often associated with the physiological roles of the enzymatic proteins [51]. The non-covalent interactions between ligands (substrates) and receptors (enzymatic proteins) mainly affect their conformation and electron distribution, which in turn affect the binding energy between them. Our results showed that AA mutations in ACAA1(S357N), ACAA2(T234I), and ACACB(H1418N) altered the non-covalent interactions between the enzyme (receptor) and its substrate (ligand), which included the loss or formation of hydrogen bonds and hydrophobic interactions, as well as changes in bonding types. In molecular docking, a binding affinity of lower than −5.0 kcal/mol is generally considered as a standard for stable ligand–receptor binding [52]. Our results demonstrated that certain changes in AAs were accompanied by a decreased ligand–receptor binding affinity. At position 357 in ACAA1, when Ser was converted to Asn, the binding energy between the ligand and receptor decreased from −7.5 kcal/mol to −5.8 kcal/mol. At position 234 in ACAA2, the conversion of Thr to Ile resulted in a decrease in the ligand–receptor binding affinity from −5.9 kcal/mol to −5.3 kcal/mol. In the case of ACACB(H1418) and ACACB(N1418), the ligand–receptor binding affinity was −5.3 kcal/mol and −5.1 kcal/mol, respectively. Although the change in the value of the binding energy itself was not very huge (in the current case 2.1, 0.6, and 0.2 kcal/mol for ACAA1, ACAA2, and ACACB, respectively), the altered binding affinity may affect the surrounding microenvironment (such as water molecules and ions) and the dynamic balance (dissociation or association) between ligands and receptors, and therefore may lead to significant alterations in the characteristics of enzyme–substrate binding. Since a stronger interaction between the ligand and the receptor could potentially lead to an improvement in the efficiency of enzymatic catalysis, AAs at these positions may be important contributors to proper substrate–enzyme interactions.

ACAA1 and ACAA2, the key enzymes responsible for the thiolytic cleavage of 3-oxoacyl-CoAs into acetyl-CoA and a fatty acyl-CoA shortened by two carbon atoms, play important roles in FA beta-oxidation in the peroxisome [53] and mitochondrion [54], respectively. In chicken intramuscular preadipocytes, *ACAA1* was the target of miR-15a. Its decreased expression reduced FA oxidation and promoted cell differentiation [55]. ACACB plays a central role in FA metabolism by catalyzing the carboxylation of acetyl-CoA to malonyl-CoA in mitochondria. Together with its cytosolic isozyme ACACA, which is involved in de novo FA biosynthesis, lipogenic ACACB promotes lipid storage [56]. *ACACB* exhibited significantly differential expression in the muscle and abdominal fat tissues in female broilers [57].

Here, in pigeons, we demonstrated for the first time that mutations occurring in three key genes, *ACAA1*, *ACAA2,* and *ACACB*, the encoding rate-limiting enzymes in FA metabolism, resulted in non-synonymous AA replacements, which affected the binding characteristics between enzymatic proteins and their substrates, and may consequently influence FA-related traits in muscles.

Our current results confirmed that SQ squabs and WK squabs displayed disparities in their IMF content and FA and AA compositions in the breast muscles. Using the ddRAD sequencing technique, we explored the genetic variants across the whole genome of pigeons. Through pathway analysis, 19 functional genes were identified as candidate genes affecting the fat-related traits of meat quality in squabs. Among them, *ACAA1*, *ACAA2*, and *ACACB* were promising functional genes, harboring SNPs causing non-synonymous AA mutations in proteins, which resulted in alterations in the non-bonding substrate–protein interactions (the Graphical Abstract). Overall, the variants identified in the present study enriched our understanding of the genetic variations in meat-type pigeons.

## 5. Conclusions

The SNPs in *ACAA1*, *ACAA2*, and *ACACB* may be referenced for developing SNP markers in future breeding programs to select squabs with optimized IMF contents and FA profiles in the breast muscles.

## Figures and Tables

**Table 1:** Physical properties and fat-related traits of the breast muscles of 4-week-old squabs.

Items	Female SQ	Female WK	*p*-Value	Male SQ	Male WK	*p*-Value
Body weight (g)	510.0 ± 5.8	444.0 ± 11.4	0.007 **	526.7 ± 8.8	470.0 ± 21.4	0.070
WBSF (Kg/f)	1.060 ± 0.045	1.080 ± 0.021	0.708	1.037 ± 0.222	1.627 ± 0.206	0.123
WHC(%)	63.59 ± 1.41	63.58 ± 0.81	0.996	61.85 ± 1.65	63.90 ± 0.82	0.328
IMF%	3.2328 ± 0.1930	2.0225 ± 0.4125	0.004	2.0384 ± 0.1636	1.7731 ± 0.3078	0.307
C16:0	22.3421 ± 0.2818	22.0875 ± 0.4921	0.677	23.6477 ± 0.4845	21.0036 ± 0.6779	0.034 *
C18:0	8.7136 ± 0.2263	10.3539 ± 0.8077	0.122	9.8626 ± 0.7161	10.3701 ± 0.7234	0.644
∑SFA	34.2023 ± 0.4369	34.2604 ± 0.4597	0.931	35.2068 ± 0.2799	33.0905 ± 0.7618	0.060
C16:1, *cis*-9	5.4010 ± 0.2921	9.009 ± 0.5994	0.006 *	5.0882 ± 0.2713	8.6849 ± 1.2331	0.046 *
C18:1, *cis*-9	32.6232 ± 0.6590	36.8815 ± 1.3255	0.045 *	32.4019 ± 1.4223	35.9913 ± 1.1260	0.119
∑MUFA	38.9323 ± 1.0076	45.8906 ± 1.7524	0.026 *	38.2960 ± 1.5041	44.7817 ± 1.9014	0.056
C18:2, *cis*-9,12	21.5117 ± 1.2321	15.2448 ± 0.9287	0.015 *	21.3717 ± 0.1038	16.8426 ± 0.9562	0.009 **
C20:3, *cis*-11,14,17	4.0916 ± 0.3035	3.9388 ± 0.3734	0.767	3.0832 ± 0.9397	4.6960 ± 0.4949	0.448
C20:5, *cis*-5,8,11,14,17	0.9758 ± 0.0618	0.5803 ± 0.0416	0.006 *	0.8500 ± 0.1770	0.6738 ± 0.1114	0.447
∑PUFA	26.8655 ± 1.1656	19.7638 ± 1.3420	0.016 *	26.3058 ± 1.2389	22.1278 ± 1.5426	0.102
∑ω-3FA	5.1711 ± 0.3757	4.5191 ± 0.4149	0.309	4.7237 ± 1.1882	5.2853 ± 0.6127	0.696
∑ω-6FA	21.6943 ± 1.2402	15.2448 ± 0.9287	0.014 *	21.5821 ± 0.1005	16.8426 ± 0.9562	0.008 **
ω-6/ω-3	4.2529 ± 0.4733	3.4069 ± 0.1122	0.157	5.5360 ± 1.8704	3.2377 ± 0.1978	0.289
P/S	0.6293 ± 0.0995	0.5220 ± 0.0105	0.344	0.7501 ± 0.0272	0.4952 ± 0.0170	0.001 **

Note: values are presented as mean ± SE; SQ, the Shiqi squabs; WK, the White King squabs; WBSF, Warner–Bratzler shear force; WHC, water-holding capacity; ∑SFA, total saturated fatty acids; ∑MUFA, total monounsaturated fatty acids; ∑PUFA, total polyunsaturated fatty acids; ∑ω-3, total omega-3 fatty acids; ∑ω-6, total omega-6 fatty acids; P/S, the ratio of polyunsaturated fatty acids to saturated fatty acids; ** and * indicate significant differences between the two breeds (*p* < 0.01 and *p* < 0.05, respectively).

**Table 2:** Composition of amino acids (AAs)% in the breast muscles of 4-week-old squabs.

		Female SQ	Female WK	*p*-Value	Male SQ	Male WK	*p*-Value
NEAA	Asp	3.40 ± 0.15	3.59 ± 0.26	0.226	3.54 ± 0.13	3.92 ± 0.12	0.001 **
	Glu	6.13 ± 0.23	6.37 ± 0.53	0.407	6.32 ± 0.29	7.06 ± 0.20	0.001 **
	Arg	1.12 ± 0.17	1.11 ± 0.17	0.932	1.20 ± 0.19	1.13 ± 0.17	0.628
	Gly	2.31 ± 0.11	2.44 ± 0.17	0.218	2.46 ± 0.12	2.71 ± 0.11	0.009 **
	Ala	2.90 ± 0.15	3.06 ± 0.23	0.299	3.07 ± 0.13	3.30 ± 0.14	0.048 *
	Ser	1.85 ± 0.10	1.94 ± 0.17	0.390	1.94 ± 0.12	2.11 ± 0.10	0.069
	Cys	0.18 ± 0.02	0.20 ± 0.05	0.587	0.18 ± 0.02	0.21 ± 0.02	0.116
	Tyr	1.73 ± 0.09	1.72 ± 0.17	0.864	1.82 ± 0.10	1.88 ± 0.08	0.360
	His	0.82 ± 0.12	0.85 ± 0.08	0.709	0.78 ± 0.12	0.87 ± 0.10	0.264
	Pro	0.68 ± 0.05	0.64 ± 0.06	0.377	0.64 ± 0.05	0.71 ± 0.06	0.079
EAA	Lys	2.11 ± 0.16	2.00 ± 0.19	0.381	2.06 ± 0.18	1.99 ± 0.19	0.628
	Val	2.70 ± 0.13	2.75 ± 0.24	0.694	2.80 ± 0.12	3.01 ± 0.08	0.014 *
	Met	0.76 ± 0.13	0.76 ± 0.14	0.977	0.77 ± 0.13	0.85 ± 0.12	0.438
	Ile	2.61 ± 0.14	2.56 ± 0.22	0.730	2.72 ± 0.15	2.87 ± 0.09	0.173
	Leu	3.07 ± 0.14	3.10 ± 0.24	0.857	3.18 ± 0.16	3.43 ± 0.10	0.024 *
	Phe	2.79 ± 0.13	2.83 ± 0.30	0.779	2.92 ± 0.14	3.12 ± 0.09	0.054
	Thr	2.22 ± 0.19	2.29 ± 0.26	0.670	2.12 ± 0.43	2.53 ± 0.20	0.146
EAA/NEAA		76.96 ± 1.28	74.08 ± 2.00	0.028 *	75.53 ± 3.06	74.51 ± 1.71	0.609

Note: Values are presented as mean ± SE; SQ, the Shiqi squabs; WK, the White King squabs; EAAs, essential amino acids; NEAAs, non-essential amino acids; ** and * indicate significant differences between the two breeds (*p* < 0.01 and *p* < 0.05, respectively).

**Table 3:** SNPs located on functional genes and resulting in non-synonymous mutations.

Reference	Gene	Position	Codon	Amino Acid (Position)	Protein ID
NW_004973530.1	*ACAA1* (*acetyl-CoA acyltransferase 1*)	195222	A**G**T/A**A**T	Ser/Asn (357)	XP_005509181.1
NW_004973534.1	*ACAA2* (*acetyl-CoA acyltransferase 2*)	468878	A**C**T/A**T**T	Thr/Ile (234)	XP_005509195.2
NW_004973182.1	*ACACB* (*acetyl-CoA carboxylase beta*)	2195260	**C**AC/**A**AC	His/Asn (1418)	XP_021140886.1
NW_004973254.1	*ACAT1* (*acetyl-CoA acetyltransferase 1*)	7307277	AA**C**/AA**A**	Asn/Lys (143)	XP_021141966.1
NW_004973187.1	*ACOX3* (*acyl-CoA oxidase 3, pristanoyl*)	1445784	**G**CC/**T**CC	Ala/Ser (329)	XP_021143932.1
NW_004973678.1	*FADS1* (*fatty acid desaturase 1*)	407943	**G**CA/**A**CA	Ala/Thr (76)	XP_005511095.1
NW_004974432.1	*HADHA* (*hydroxyacyl-CoA dehydrogenase, alpha subunit*)	29797	**G**AG/**A**AG	Glu/Lys (314)	XP_021138551.1
NW_004974432.1	*HADHB* (*hydroxyacyl-CoA dehydrogenase, beta subunit*)	20595	**A**AA/**G**AA	Lys/Glu (96)	XP_013226901.1
NW_004973569.1	*MECR* (*mitochondrial trans-2-enoyl-CoA reductase*)	3420884	A**C**G/A**T**G	Thr/Met (218)	XP_021153195.1
NW_004973196.1	*OXSM* (*3-oxoacyl-ACP synthase, mitochondrial*)	1164225	C**A**C/C**G**C	His/Arg (5)	XP_005499238.1

**Table 4:** Clustering analysis of protein–protein interaction relationships of the 19 functional genes.

Cluster	Description	Strength	lgFDR
CL:20682	acetyl-CoA C-acyltransferase activity and fatty acid beta-oxidation using acyl-CoA dehydrogenase	2.65	−6.75
CL:20720	decanoate-CoA ligase activity and isobutyryl-CoA dehydrogenase	2.59	−6.61
CL:20806	Fatty acid biosynthesis	2.49	−2.14
CL:20660	Fatty acid beta-oxidation	2.41	−10.08
CL:20663	Acyl-CoA oxidase and MaoC-like domain	2.41	−2.04
CL:20652	Fatty acid beta-oxidation and medium-chain fatty acid-CoA ligase activity	2.35	−22.43
CL:20655	Fatty acid beta-oxidation and decanoate-CoA ligase activity	2.33	−13.67
CL:20650	Fatty acid beta-oxidation and medium-chain fatty acid-CoA ligase activity	2.26	−23.73
CL:20963	Fatty acid biosynthesis	2.19	−1.70
CL:20961	Biosynthesis of unsaturated fatty acids	2.16	−3.39
CL:20648	Valine, leucine, and isoleucine degradation and fatty acid metabolism	2.15	−28.75
CL:20649	Valine, leucine, and isoleucine degradation and fatty acid beta-oxidation	2.14	−24.44
CL:20644	Fatty acid metabolic process and microbody membrane	1.92	−32.47

## Data Availability

Raw sequence data obtained in the ddRAD sequencing have been deposited in the NCBI Sequence Read Archive (SRA) under the BioProject ID PRJNA982969.

## Supplementary Materials

The following supporting information can be downloaded at https://www.mdpi.com/article/10.3390/ani13203256/s1, Table S1: Basic information of sequencing data and the quality; Table S2: Details of the mapping results; Table S3: The resolved center coordinate used in molecular docking; Table S4: Studies regarding the identification of candidate genes associated with fat-related traits in livestock. References [53,54,56,58,59,60,61,62,63,64,65,66,67,68,69,70,71] are cited in the Supplementary Materials.

## References

[1] Jiang S.G., Pan N.X., Chen M.J., Wang X.Q., Yan H.C., Gao C.Q. (2019). Effects of dietary supplementation with dl-methionine and dl-methionyl-dl-methionine in breeding pigeons on the carcass characteristics, meat quality and antioxidant activity of squabs. Antioxidants.

[2] Yin Z., Zhou W., Mao H., Dong X., Huang X., Zhang H., Liu H. (2022). Identification of genes related to squab muscle growth and lipid metabolism from transcriptome profiles of breast muscle and liver in domestic pigeon (*Columba livia*). Animals.

[3] Huang Y., Liu L., Zhao M., Zhang X., Chen J., Zhang Z., Cheng X., Ren C. (2023). Feeding regimens affecting carcass and quality attributes of sheep and goat meat: A comprehensive review. Anim. Biosci..

[4] Estany J., Ros-Freixedes R., Tor M., Pena R.N. (2017). Triennial growth and development symposium: Genetics and breeding for intramuscular fat and oleic acid content in pigs. J. Anim. Sci..

[5] Baik M., Kang H.J., Park S.J., Na S.W., Piao M., Kim S.Y., Fassah D.M., Moon Y.S. (2017). Triennial growth and development symposium: Molecular mechanisms related to bovine intramuscular fat deposition in the *Longissimus* muscle. J. Anim. Sci..

[6] Mir N.A., Rafiq A., Kumar F., Singh V., Shukla V. (2017). Determinants of broiler chicken meat quality and factors affecting them: A review. J. Food Sci. Technol..

[7] Lin Y., Zhu J., Wang Y., Li Q., Lin S. (2017). Identification of differentially expressed genes through RNA sequencing in goats (*Capra hircus*) at different postnatal stages. PLoS ONE.

[8] Li G., Yang R., Lu X., Liu Y., He W., Li Y., Yu H., Qin L., Cao Y., Zhao Z. (2022). RNA-seq analysis identifies differentially expressed genes in the *Longissimus dorsi* of wagyu and chinese red steppe cattle. Int. J. Mol. Sci..

[9] Zhao L., Zhou L., Hao X., Wang L., Han F., Liu L., Duan X., Guo F., He J., Liu N. (2021). Identification and characterization of circular rnas in association with the deposition of intramuscular fat in aohan fine-wool sheep. Front. Genet..

[10] Wang Y.L., Hou Y.H., Ling Z.J., Zhao H.L., Zheng X.R., Zhang X.D., Yin Z.J., Ding Y.Y. (2023). RNA sequencing analysis of the *Longissimus dorsi* to identify candidate genes underlying the intramuscular fat content in Anqing Six-end-white pigs. Anim. Genet..

[11] Kang H., Zhao D., Xiang H., Li J., Zhao G., Li H. (2021). Large-scale transcriptome sequencing in broiler chickens to identify candidate genes for breast muscle weight and intramuscular fat content. Genet. Sel. Evol..

[12] Wang L., Li X., Ma J., Zhang Y., Zhang H. (2017). Integrating genome and transcriptome profiling for elucidating the mechanism of muscle growth and lipid deposition in Pekin ducks. Sci. Rep..

[13] Ye M., Zhou B., Wei S., Ding M., Lu X., Shi X., Ding J., Yang S., Wei W. (2016). Transcriptomic analysis identifies candidate genes related to intramuscular fat deposition and fatty acid composition in the breast muscle of squabs (*Columba*). G3 (Bethesda).

[14] Dong X., Cao H., Mao H., Hong Q., Yin Z. (2019). Association of *MyoD1* gene polymorphisms with meat quality traits in domestic pigeons (*Columba livia*). J. Poult. Sci..

[15] Mao H.G., Xu X.L., Cao H.Y., Dong X.Y., Zou X.T., Xu N.Y., Yin Z.Z. (2021). *H-FABP* gene expression and genetic association with meat quality traits in domestic pigeons (*Columba livia*). Br. Poult. Sci..

[16] Masharing N., Sodhi M., Chanda D., Singh I., Vivek P., Tiwari M., Kumari P., Mukesh M. (2023). ddRAD sequencing based genotyping of six indigenous dairy cattle breeds of India to infer existing genetic diversity and population structure. Sci. Rep..

[17] Rahman J.U., Kumar D., Singh S.P., Shahi B.N., Ghosh A.K., Verma M.K., Pathak A., Dar A.H., Kumar A., Sharma R.K. (2023). Genome-wide identification and annotation of SNPs and their mapping in candidate genes related to milk production and fertility traits in Badri cattle. Trop. Anim. Health Prod..

[18] Raja T.V., Alex R., Singh U., Kumar S., Das A.K., Sengar G., Singh A.K. (2023). Genome wide mining of SNPs and INDELs through ddRAD sequencing in Sahiwal cattle. Anim. Biotechnol..

[19] Kour A., Niranjan S.K., Malayaperumal M., Surati U., Pukhrambam M., Sivalingam J., Kumar A., Sarkar M. (2022). Genomic diversity profiling and breed-specific evolutionary signatures of selection in arunachali yak. Genes.

[20] Ye M., Xu M., Lu M., Zhou B., Heba A.E.K., Said A.S., Fathy M.K. (2020). Identification of candidate genes associated with milk yield trait in buffaloes (*Bubalus bubalis*) by restriction-site-associated DNA sequencing. R. Bras. Zootec..

[21] Chen Q., Ma Y., Yang Y., Chen Z., Liao R., Xie X., Wang Z., He P., Tu Y., Zhang X. (2013). Genotyping by genome reducing and sequencing for outbred animals. PLoS ONE.

[22] Herry F., Hérault F., Lecerf F., Lagoutte L., Doublet M., Picard-Druet D., Bardou P., Varenne A., Burlot T., Le Roy P. (2023). Restriction site-associated DNA sequencing technologies as an alternative to low-density SNP chips for genomic selection: A simulation study in layer chickens. BMC Genom..

[23] Zhai Z., Zhao W., He C., Yang K., Tang L., Liu S., Zhang Y., Huang Q., Meng H. (2015). SNP discovery and genotyping using restriction-site-associated DNA sequencing in chickens. Anim. Genet..

[24] Liao R., Wang Z., Chen Q., Tu Y., Chen Z., Wang Q., Yang C., Zhang X., Pan Y. (2015). An efficient genotyping method in chicken based on genome reducing and sequencing. PLoS ONE.

[25] Pértille F., Guerrero-Bosagna C., Silva V.H., Boschiero C., Nunes J.R., Ledur M.C., Jensen P., Coutinho L.L. (2016). High-throughput and cost-effective chicken genotyping using next-generation sequencing. Sci. Rep..

[26] Ye M., Xu M., Chen C., He Y., Ding M., Ding X., Wei W., Yang S., Zhou B. (2018). Expression analyses of candidate genes related to meat quality traits in squabs from two breeds of meat-type pigeon. J. Anim. Physiol. Anim. Nutr..

[27] Mohanty M., Mohanty P.S. (2023). Molecular docking in organic, inorganic, and hybrid systems: A tutorial review. Monatsh. Chem..

[28] Callil-Soares P.H., Biasi L.C.K., Pessoa Filho P.A. (2023). Effect of preprocessing and simulation parameters on the performance of molecular docking studies. J. Mol. Model..

[29] Peterson B.K., Weber J.N., Kay E.H., Fisher H.S., Hoekstra H.E. (2012). Double digest RADseq: An inexpensive method for de novo SNP discovery and genotyping in model and non-model species. PLoS ONE.

[30] Bolger A.M., Lohse M., Usadel B. (2014). Trimmomatic: A flexible trimmer for Illumina sequence data. Bioinformatics.

[31] Rochette N.C., Rivera-Colón A.G., Catchen J.M. (2019). Stacks 2: Analytical methods for paired-end sequencing improve RADseq-based population genomics. Mol. Ecol..

[32] Li H., Durbin R. (2009). Fast and accurate short read alignment with Burrows-Wheeler transform. Bioinformatics.

[33] Van der Auwera G.A., Carneiro M.O., Hartl C., Poplin R., Del Angel G., Levy-Moonshine A., Jordan T., Shakir K., Roazen D., Thibault J. (2013). From FastQ data to high confidence variant calls: The Genome Analysis Toolkit best practices pipeline. Curr. Protoc. Bioinform..

[34] Danecek P., Auton A., Abecasis G., Albers C.A., Banks E., DePristo M.A., Handsaker R.E., Lunter G., Marth G.T., Sherry S.T. (2011). The variant call format and VCFtools. Bioinformatics.

[35] Trott O., Olson A.J. (2010). AutoDock Vina: Improving the speed and accuracy of docking with a new scoring function, efficient optimization, and multithreading. J. Comput. Chem..

[36] Peng J., Huang W., Liang Y., Zhang W., Zhang Y., Yang M., Zheng S., Lv Y., Gou Z., Cheng C. (2023). Optimal dietary energy and protein levels for breeding pigeons in the winter “2 + 3” lactation pattern. Poult. Sci..

[37] Peng J., Huang W., Zhang W., Zhang Y., Yang M., Zheng S., Lv Y., Gao H., Wang W., Peng J. (2023). Effect of different dietary energy/protein ratios on growth performance, reproductive performance of breeding pigeons and slaughter performance, meat quality of squabs in summer. Poult. Sci..

[38] Zhang R., Ma H., Han P., Li Y., Sun Y., Yuan J., Wang Y., Ni A., Zong Y., Bian S. (2022). Effects of feed systems on growth performance, carcass characteristics, organ index, and serum biochemical parameters of pigeon. Poult. Sci..

[39] Liu T., Wang L., Jiang X., Liu Y., Diao E., Xie P. (2023). Free-choice feeding of whole grains improves meat quality and intestinal development of pigeon squabs compared with complete pelleted feed. Life.

[40] Xu Q., Wang X., Liu Y., Dong X., Zou X. (2021). Parental dietary arachidonic acid altered serum fatty acid profile, hepatic antioxidant capacity, and lipid metabolism in domestic pigeons (*Columba livia*). Anim. Sci. J..

[41] Wen J., Zhao W., Li J., Hu C., Zou X., Dong X. (2022). Dietary supplementation of chitosan oligosaccharide-clostridium *butyricum* synbiotic relieved early-weaned stress by improving intestinal health on pigeon squabs (*Columba livia*). Front. Immunol..

[42] Amer H.Y., Hassan R.I.M., Mustafa F.E.A., El-Shoukary R.D., Rehan I.F., Zigo F., Lacková Z., Gomaa W.M.S. (2023). Modulation of immunity, antioxidant status, performance, blood hematology, and intestinal histomorphometry in response to dietary inclusion of *Origanum majorana* in domestic pigeons’ diet. Life.

[43] Chang L., Tang Q., Zhang R., Fu S., Mu C., Shen X., Bu Z. (2023). Evaluation of meat quality of local pigeon varieties in China. Animals.

[44] Boschetti E., Bordoni A., Meluzzi A., Castellini C., Dal Bosco A., Sirri F. (2016). Fatty acid composition of chicken breast meat is dependent on genotype-related variation of *FADS1* and *FADS2* gene expression and desaturating activity. Animal.

[45] Li Y., Mei H., Liu Y., Li Z., Qamar H., Yu M., Ma X. (2023). Dietary supplementation with rutin alters meat quality, fatty acid profile, antioxidant capacity, and expression levels of genes associated with lipid metabolism in breast muscle of qingyuan partridge chickens. Foods.

[46] Luo N., Shu J., Yuan X., Jin Y., Cui H., Zhao G., Wen J. (2022). Differential regulation of intramuscular fat and abdominal fat deposition in chickens. BMC Genom..

[47] Chang T.Y., Li B.L., Chang C.C., Urano Y. (2009). Acyl-coenzyme A: Cholesterol acyltransferases. Am. J. Physiol. Endocrinol. Metab..

[48] Liu L., Cui H., Fu R., Zheng M., Liu R., Zhao G., Wen J. (2017). The regulation of IMF deposition in pectoralis major of fast- and slow- growing chickens at hatching. J. Anim. Sci. Biotechnol..

[49] Zhang M., Li F., Ma X.F., Li W.T., Jiang R.R., Han R.L., Li G.X., Wang Y.B., Li Z.Y., Tian Y.D. (2019). Identification of differentially expressed genes and pathways between intramuscular and abdominal fat-derived preadipocyte differentiation of chickens in vitro. BMC Genom..

[50] Zhang J., Zhuang H., Cao J., Geng A., Wang H., Chu Q., Yan Z., Zhang X., Zhang Y., Liu H. (2022). Breast meat fatty acid profiling and proteomic analysis of Beijing-You chicken during the laying period. Front. Vet. Sci..

[51] Dutta D., Mishra S. (2016). Structural and mechanistic insight into substrate binding from the conformational dynamics in apo and substrate-bound DapE enzyme. Phys. Chem. Chem. Phys..

[52] Xu Z., Shen M., Li L. (2023). Exploring the active components and mechanism of modified bazhen decoction in treatment of chronic cerebral circulation insufficiency based on network pharmacology and molecular docking. Medicine.

[53] Ferdinandusse S., Denis S., Mooijer P.A., Zhang Z., Reddy J.K., Spector A.A., Wanders R.J. (2001). Identification of the peroxisomal beta-oxidation enzymes involved in the biosynthesis of docosahexaenoic acid. J. Lipid Res..

[54] Kiema T.R., Harijan R.K., Strozyk M., Fukao T., Alexson S.E., Wierenga R.K. (2014). The crystal structure of human mitochondrial 3-ketoacyl-CoA thiolase (T1): Insight into the reaction mechanism of its thiolase and thioesterase activities. Acta Crystallogr. D Biol. Crystallogr..

[55] Li G., Fu S., Chen Y., Jin W., Zhai B., Li Y., Sun G., Han R., Wang Y., Tian Y. (2019). MicroRNA-15a regulates the differentiation of intramuscular preadipocytes by targeting *ACAA1*, *ACOX1* and *SCP2* in chickens. Int. J. Mol. Sci..

[56] Harriman G., Greenwood J., Bhat S., Huang X., Wang R., Paul D., Tong L., Saha A.K., Westlin W.F., Kapeller R. (2016). Acetyl-CoA carboxylase inhibition by ND-630 reduces hepatic steatosis, improves insulin sensitivity, and modulates dyslipidemia in rats. Proc. Natl. Acad. Sci. USA.

[57] Zhang M., Zheng D., Peng Z., Zhu Y., Li R., Wu Q., Li Y., Li H., Xu W., Zhang M. (2021). Identification of differentially expressed genes and lipid metabolism signaling pathways between muscle and fat tissues in broiler chickens. J. Poult. Sci..

[58] Luo C., Zhao S., Dai W., Zheng N., Wang J. (2018). Proteomic analysis of lysosomal membrane proteins in bovine mammary epithelial cells illuminates potential novel lysosome functions in lactation. J. Agric. Food Chem..

[59] Wang Y., Li X., Cao Y., Xiao C., Liu Y., Jin H., Cao Y. (2021). Effect of the *ACAA1* gene on preadipocyte differentiation in sheep. Front. Genet..

[60] Miltiadou D., Hager-Theodorides A.L., Symeou S., Constantinou C., Psifidi A., Banos G., Tzamaloukas O. (2017). Variants in the 3′ untranslated region of the ovine acetyl-coenzyme A acyltransferase 2 gene are associated with dairy traits and exhibit differential allelic expression. J. Dairy Sci..

[61] Symeou S., Tzamaloukas O., Banos G., Miltiadou D. (2020). *ACAA2* and *FASN* polymorphisms affect the fatty acid profile of Chios sheep milk. J. Dairy Res..

[62] Han B., Liang W., Liu L., Li Y., Sun D. (2018). Genetic association of the *ACACB* gene with milk yield and composition traits in dairy cattle. Anim. Genet..

[63] Wang X., Zhang Y., Zhang X., Wang D., Jin G., Li B., Xu F., Cheng J., Zhang F., Wu S. (2017). The comprehensive liver transcriptome of two cattle breeds with different intramuscular fat content. Biochem. Biophys. Res. Commun..

[64] Wang L., Li J., Hou X., Yan H., Zhang L., Liu X., Gao H., Zhao F., Wang L. (2020). Genome-wide identification of RNA editing sites affecting intramuscular fat in pigs. Animals.

[65] Silva-Vignato B., Coutinho L.L., Poleti M.D., Cesar A.S.M., Moncau C.T., Regitano L.C.A., Balieiro J.C.C. (2019). Gene co-expression networks associated with carcass traits reveal new pathways for muscle and fat deposition in Nelore cattle. BMC Genom..

[66] Zappaterra M., Luise D., Zambonelli P., Mele M., Serra A., Costa L.N., Davoli R. (2019). Association study between backfat fatty acid composition and SNPs in candidate genes highlights the effect of *FASN* polymorphism in large white pigs. Meat Sci..

[67] Corominas J., Ramayo-Caldas Y., Puig-Oliveras A., Pérez-Montarelo D., Noguera J.L., Folch J.M., Ballester M. (2013). Polymorphism in the *ELOVL6* gene is associated with a major QTL effect on fatty acid composition in pigs. PLoS ONE.

[68] Crespo-Piazuelo D., Criado-Mesas L., Revilla M., Castelló A., Noguera J.L., Fernández A.I., Ballester M., Folch J.M. (2020). Identification of strong candidate genes for backfat and intramuscular fatty acid composition in three crosses based on the Iberian pig. Sci. Rep..

[69] Palma-Granados P., García-Casco J.M., Caraballo C., Vázquez-Ortego P., Gómez-Carballar F., Sánchez-Esquiliche F., Óvilo C., Muñoz M. (2023). Design of a low-density SNP panel for intramuscular fat content and fatty acid composition of backfat in free-range Iberian pigs. J. Anim. Sci..

[70] Xie T., Liu Y., Lu H., Iqbal A., Ruan M., Jiang P., Yu H., Meng J., Zhao Z. (2022). The knockout of the *ASIP* gene altered the lipid composition in bovine mammary epithelial cells via the expression of genes in the lipid metabolism pathway. Animals.

[71] Wang S., Yang C., Pan C., Feng X., Lei Z., Huang J., Wei X., Li F., Ma Y. (2022). Identification of key genes and functional enrichment pathways involved in fat deposition in Xinyang buffalo by WGCNA. Gene.

