# Supplementary material for: The Identification of Functional Genes Affecting Fat-Related Meat Traits in Meat-Type Pigeons Using Double-Digest Restriction-Associated DNA Sequencing and Molecular Docking Analysis
Source: Animals (Basel). 2023 Oct 19;13(20):3256. doi: 10.3390/ani13203256 (PMC10603692; doi:10.3390/ani13203256)
Supplement: Supplementary file 1 [file animals-13-03256-s001.zip › Table S1.pdf]

Table S1. Basic information of sequencing data and the quality.

ID	Sex	Raw reads number	Base number	Reads length (bp)	GC (%)	Q30 (%)	Q20 (%)	Average Q (%)
SQ1	Female	10,882,608	1,605,184,680	148	43.74	82.74	91.8	36.06
SQ2	Female	6,801,306	1,001,803,745	147	42.41	84.99	93.01	36.56
SQ3	Female	10,561,462	1,557,815,645	148	44.43	83.71	92.45	36.34
SQ4	Male	9,469,698	1,396,338,730	147	42.57	86.82	93.93	36.98
SQ5	Male	13,454,166	1,991,216,568	148	45.79	83.3	92.17	36.2
SQ6	Male	6,938,090	1,023,018,652	147	42.50	86.96	94.04	37.02
Average for SQ		9,684,555	1,429,229,670	147	43.57	84.75	92.90	36.53
WK1	Female	8,954,878	1,316,367,066	147	44.38	85.87	93.55	36.74
WK2	Female	12,031,918	1,768,691,946	147	46.90	83.25	92.19	36.19
WK3	Female	5,779,428	854,025,440	148	44.42	84.81	93.01	36.56
WK4	Male	8,412,672	1,236,662,784	147	46.82	82.88	91.92	36.09
WK5	Male	8,637,990	1,269,784,530	147	45.83	83.4	92.27	36.24
WK6	Male	9,977,442	1,466,683,974	147	45.95	83.29	92.18	36.21
Average for WK		8,965,721	1,318,702,623	147	45.72	83.92	92.52	36.34

Note: SQ1-SQ6 and WK1-WK6 are from Shiqi (SQ) and White king (WK) squabs, respectively.
