# Supplementary material for: The Identification of Functional Genes Affecting Fat-Related Meat Traits in Meat-Type Pigeons Using Double-Digest Restriction-Associated DNA Sequencing and Molecular Docking Analysis
Source: Animals (Basel). 2023 Oct 19;13(20):3256. doi: 10.3390/ani13203256 (PMC10603692; doi:10.3390/ani13203256)
Supplement: Supplementary file 1 [file animals-13-03256-s001.zip › Table S2.pdf]

Table S2. Details of the mapping results.

ID	Sex	Mapped paired reads	Mapping rate (%)	Cover size (bp)	Average coverage depth	Average genome depth	Coverage (1×)	Coverage (5×)	Coverage (10×)
SQ1	Female	10579050	98.48	227384603	6.95	1.43	20.52	6.62	3.68
SQ2	Female	6650584	98.68	153748745	6.43	0.89	13.88	5.28	2.47
SQ3	Female	10281424	98.56	213820516	7.18	1.39	19.30	5.24	3.57
SQ4	Male	9272102	98.77	201219081	6.85	1.24	18.16	6.57	3.59
SQ5	Male	13040446	98.39	245022691	7.99	1.77	22.11	6.49	3.94
SQ6	Male	6809696	98.88	162192266	6.24	0.91	14.64	5.30	2.47
Average of SQ		9438884	98.63	200564650	6.94	1.27	18.10	5.92	3.29
WK1	Female	8748366	98.62	161558136	8.03	1.17	14.58	4.03	2.69
WK2	Female	11659850	98.34	218160442	7.97	1.57	19.69	5.66	3.14
WK3	Female	5629370	98.46	135469813	6.21	0.76	12.23	3.49	2.06
WK4	Male	8160474	98.41	194989072	6.24	1.10	17.60	4.42	2.12
WK5	Male	8402736	98.54	194880115	6.42	1.13	17.59	4.69	2.75
WK6	Male	9699328	98.50	201148795	7.18	1.30	18.15	5.01	3.02
Average of WK		8716687	98.48	184367729	7.01	1.17	16.64	4.55	2.63

Note: The genome size of pigeon (*Columba livia*) is 1,107,989,085 bp.
