# Supplementary material for: The Identification of Functional Genes Affecting Fat-Related Meat Traits in Meat-Type Pigeons Using Double-Digest Restriction-Associated DNA Sequencing and Molecular Docking Analysis
Source: Animals (Basel). 2023 Oct 19;13(20):3256. doi: 10.3390/ani13203256 (PMC10603692; doi:10.3390/ani13203256)
Supplement: Supplementary file 1 [file animals-13-03256-s001.zip › Table S3.pdf]

Table S3. The resolved center coordinate used in molecular docking.

		ACAA1	ACAA2	ACACB
Ligand		coenzyme A	myristoyl-CoA	acetyl-CoA
CHEBI		57287 [1]	57385 [2]	57288 [3]
Center (Angstrom)	x	-11.865	-14.643	294.018
	y	7.030	16.877	234.668
	z	-0.436	10.835	120.720
Dimensions (Angstrom)	x	82.158	109.850	135.869
	y	82.5225	91.131	185.534
	z	92.7045	89.528	150.164

Note: ACAA1, acetyl-CoA acyltransferase 1; ACAA2, acetyl-CoA acyltransferase 2; ACACB, acetyl-CoA carboxylase beta.

Reference

1. Ferdinandusse, S.; Denis, S.; Mooijer, P.A.; Zhang, Z.; Reddy, J.K.; Spector, A.A.; Wanders, R.J. Identification of the peroxisomal beta-oxidation enzymes involved in the biosynthesis of docosahexaenoic acid. *J. Lipid Res*. **2001**, *42*, 1987-95. doi: 10.1016/S0022-2275(20)31527-3
2. Kiema, T.R.; Harijan, R.K.; Strozyk, M.; Fukao, T.; Alexson, S.E.; Wierenga, R.K. The crystal structure of human mitochondrial 3-ketoacyl-CoA thiolase (T1): insight into the reaction mechanism of its thiolase and thioesterase activities. *Acta Crystallogr. D Biol. Crystallogr*. **2014**, *70*, 3212-25. doi: 10.1107/S1399004714023827
3. Harriman, G.; Greenwood, J.; Bhat, S.; Huang, X.; Wang, R.; Paul, D.; Tong, L.; Saha, A.K.; Westlin, W.F.; Kapeller, R.; Harwood, HJ. Jr. Acetyl-CoA carboxylase inhibition by ND-630 reduces hepatic steatosis, improves insulin sensitivity, and modulates dyslipidemia in rats. *Proc. Natl. Acad. Sci. USA*. **2016**, *113*, E1796-805. doi: 10.1073/pnas.1520686113
