# Supplementary material for: The Identification of Functional Genes Affecting Fat-Related Meat Traits in Meat-Type Pigeons Using Double-Digest Restriction-Associated DNA Sequencing and Molecular Docking Analysis
Source: Animals (Basel). 2023 Oct 19;13(20):3256. doi: 10.3390/ani13203256 (PMC10603692; doi:10.3390/ani13203256)
Supplement: Supplementary file 1 [file animals-13-03256-s001.zip › Table S4.pdf]

Table S4. Studies regarding the identification of candidate genes associated with fat-related traits in livestock.

Gene	Species/cells	Association with fat-related traits
ACAA1	Bovine mammary epithelial cells	Involved in milk fat synthesis during the lactation process [1]
ACAA1	Sheep preadipocytes	The deficiency of *ACAA1* promoted adipogenesis and lipid accumulation, while its overexpression inhibited cell differentiation [2]
ACAA2	Sheep	A SNP on the 3′-UTR (a T to C transition) of *ACAA2* was associated milk yield, milk protein percentage, fatty acid profile, and the ω-6/ω-3 ratio in the milk of ewes [3-4]
ACACB	Cattle	SNPs in *ACACB* were significantly associated with fat yield and fat percentage of the milk in a Chinese Holstein population [5]
ACACB	Cattle	Significantly differentially expressed in two cattle populations showing disparities in intramuscular fat content [6]
ACAT1	Pig	Harbored candidate RNA editing sites associated with intramuscular fat content [7]
ACAT1 and *ACSL1*	Cattle	Candidate genes affecting ribeye area and backfat thickness [8]
ELOVL6	Pig	Significantly associated with fatty acid contents and proportions of fatty acids in backfat, especially palmitic acid (C16:0), palmitoleic acid (C16:1), and oleic acid (C18:1) [9-12]
ELOVL6, *ACSL1*, and *HACD4*	Bovine mammary epithelial cells	Significantly increased expression was demonstrated to promote the synthesis of mid- to long-chain fatty acids [13]
MECR	Buffalo	Associated with fat deposition and intra-muscular amino acid levels [14]

1. Luo, C.; Zhao, S.; Dai, W.; Zheng, N.; Wang, J. Proteomic analysis of lysosomal membrane proteins in bovine mammary epithelial cells illuminates potential novel lysosome functions in lactation. *J. Agric. Food Chem*. **2018**, *66*, 13041-13049. doi: 10.1021/acs.jafc.8b04508
2. Wang, Y.; Li, X.; Cao, Y.; Xiao, C.; Liu, Y.; Jin, H.; Cao, Y. Effect of the *ACAA1* gene on preadipocyte differentiation in sheep. *Front. Genet*. **2021**, *12*, 649140. doi: 10.3389/fgene.2021.649140
3. Miltiadou, D.; Hager-Theodorides, A.L.; Symeou, S.; Constantinou, C.; Psifidi, A.; Banos, G.; Tzamaloukas, O. Variants in the 3' untranslated region of the ovine acetyl-coenzyme A acyltransferase 2 gene are associated with dairy traits and exhibit differential allelic expression. *J. Dairy Sci*. **2017**, *100*, 6285-6297. doi: 10.3168/jds.2016-12326
4. Symeou, S.; Tzamaloukas, O.; Banos, G.; Miltiadou, D. *ACAA2* and *FASN* polymorphisms affect the fatty acid profile of Chios sheep milk. *J. Dairy Res*. **2020**, *87*, 23-26. doi: 10.1017/S0022029919000992
5. Han, B.; Liang, W.; Liu, L.; Li, Y.; Sun, D. Genetic association of the *ACACB* gene with milk yield and composition traits in dairy cattle. *Anim. Genet*. **2018**, *49*, 169-177. doi: 10.1111/age.12651
6. Wang, X.; Zhang, Y.; Zhang, X.; Wang, D.; Jin, G.; Li, B.; Xu, F.; Cheng, J.; Zhang, F.; Wu, S.; Rui, S.; He, J.; Zhang, R.; Liu, W. The comprehensive liver transcriptome of two cattle breeds with different intramuscular fat content. *Biochem. Biophys. Res. Commun.* **2017**, *490*. 1018-1025. doi: 10.1016/j.bbrc.2017.06.157

7. Wang, L.; Li, J.; Hou, X.; Yan, H.; Zhang, L.; Liu, X.; Gao, H.; Zhao, F.; Wang, L. Genome-wide identification of RNA editing sites affecting intramuscular fat in pigs. *Animals (Basel)*. **2020**, *10*, 1616. doi: 10.3390/ani10091616
8. Silva-Vignato, B.; Coutinho, L.L.; Poleti, M.D.; Cesar, A.S.M.; Moncau, C.T.; Regitano, L.C.A.; Balieiro, J.C.C. Gene co-expression networks associated with carcass traits reveal new pathways for muscle and fat deposition in Nelore cattle. *BMC Genomics*. **2019**, *20*, 32. doi: 10.1186/s12864-018-5345-y
9. Zappaterra, M.; Luise, D.; Zambonelli, P.; Mele, M.; Serra, A.; Costa, L.N.; Davoli, R. Association study between backfat fatty acid composition and SNPs in candidate genes highlights the effect of *FASN* polymorphism in large white pigs. *Meat Sci*. **2019**, *156*, 75-84. doi: 10.1016/j.meatsci.2019.05.013
10. Corominas, J.; Ramayo-Caldas, Y.; Puig-Oliveras, A.; Pérez-Montarelo, D.; Noguera, J.L.; Folch, J.M.; Ballester, M. Polymorphism in the *ELOVL6* gene is associated with a major QTL effect on fatty acid composition in pigs. *PLoS One*. **2013**, *8*, e53687. doi: 10.1371/journal.pone.0053687
11. Crespo-Piazuelo, D.; Criado-Mesas, L.; Revilla, M.; Castelló, A.; Noguera, J.L.; Fernández, A.I.; Ballester, M.; Folch, J.M. Identification of strong candidate genes for backfat and intramuscular fatty acid composition in three crosses based on the Iberian pig. *Sci. Rep.* **2020**, *10*, 13962. doi: 10.1038/s41598-020-70894-2
12. Palma-Granados, P.; García-Casco, J.M.; Caraballo, C.; Vázquez-Ortego, P.; Gómez-Carballar, F.; Sánchez-Esquiliche, F.; Óvilo, C.; Muñoz, M. Design of a low-density SNP panel for intramuscular fat content and fatty acid composition of backfat in free-range Iberian pigs. *J. Anim. Sci*. **2023**, *101*, skad079. doi: 10.1093/jas/skad079.
13. Xie, T.; Liu, Y.; Lu, H.; Iqbal, A.; Ruan, M.; Jiang, P.; Yu, H.; Meng, J.; Zhao, Z. The knockout of the *ASIP* gene altered the lipid composition in bovine mammary epithelial cells via the expression of genes in the lipid metabolism pathway. *Animals (Basel)*. **2022**, *12*, 1389. doi: 10.3390/ani12111389
14. Wang, S.; Yang, C.; Pan, C.; Feng, X.; Lei, Z.; Huang, J.; Wei, X.; Li, F.; Ma, Y. Identification of key genes and functional enrichment pathways involved in fat deposition in Xinyang buffalo by WGCNA. *Gene*. **2022**, *818*, 146225. doi: 10.1016/j.gene.2022.146225
